# What Are the Drivers Triggering Antimicrobial Resistance Emergence and Spread? Outlook from a One Health Perspective

**DOI:** 10.3390/antibiotics14060543

**Published:** 2025-05-26

**Authors:** Zehong Ye, Menghan Li, Yiwen Jing, Kejun Liu, Yongning Wu, Zixin Peng

**Affiliations:** 1 School of Public Health, Shandong Second Medical University, Weifang 261053, China; 2 NHC Key Laboratory of Food Safety Risk Assessment, Chinese Academy of Medical Science Research Unit (2019RU014), China National Center for Food Safety Risk Assessment, Beijing 100021, China; 3 China National Health Development Research Center, Beijing 100044, China

**Keywords:** One Health, antimicrobial resistance, antimicrobial resistance genes, driving factors

## Abstract

Antimicrobial resistance (AMR) has emerged as a critical global public health threat, exacerbating healthcare burdens and imposing substantial economic costs. Currently, AMR contributes to nearly five million deaths annually worldwide, surpassing mortality rates of any single infectious disease. The economic burden associated with AMR-related disease management is estimated at approximately $730 billion per year. This review synthesizes current research on the mechanisms and multifaceted drivers of AMR development and dissemination through the lens of the One Health framework, which integrates human, animal, and environmental health perspectives. Intrinsic factors, including antimicrobial resistance genes (ARGs) and mobile genetic elements (MGEs), enable bacteria to evolve adaptive resistance mechanisms such as enzymatic inactivation, efflux pumps, and biofilm formation. Extrinsic drivers span environmental stressors (e.g., antimicrobials, heavy metals, disinfectants), socioeconomic practices, healthcare policies, and climate change, collectively accelerating AMR proliferation. Horizontal gene transfer and ecological pressures further facilitate the spread of antimicrobial-resistant bacteria across ecosystems. The cascading impacts of AMR threaten human health and agricultural productivity, elevate foodborne infection risks, and impose substantial economic burdens, particularly in low- and middle-income countries. To address this complex issue, the review advocates for interdisciplinary collaboration, robust policy implementation (e.g., antimicrobial stewardship), and innovative technologies (e.g., genomic surveillance, predictive modeling) under the One Health paradigm. Such integrated strategies are essential to mitigate AMR transmission, safeguard global health, and ensure sustainable development.

## 1. Introduction

Bacteria have existed on Earth for billions of years and are widely distributed across various environments, including soil, water bodies, air, and within the bodies of animals and humans. Pathogenic bacteria cause diverse diseases in humans, ranging from allergic syndromes to superficial, scarring, and life-threatening invasive bacterial diseases, which together affect more than a billion people per annum worldwide [1].

Antimicrobials, encompassing antibiotics, antivirals, antifungals, and antiparasitics, constitute a class of pharmaceutical agents employed for preventing and treating infectious diseases, spanning human medicine, veterinary practice, and agricultural applications [2]. In this review, the term “antimicrobials” specifically refers to antibiotics. The discovery of penicillin revolutionized the treatment of infectious diseases, marking a pivotal advancement in medical history. However, antimicrobial therapy is not universally effective. There are also failures of antimicrobial treatment of human clinical infections [3], mainly due to (i) the immunodeficiency of the patient, (ii) the characteristics of antimicrobials (pharmacokinetics, pharmacodynamics, and drug–drug interactions, etc.), and (iii) the characteristics of bacterial biology (e.g., different cellular morphologies, drug resistance, etc.). Antimicrobial resistance (AMR) is a major global challenge to public health, food safety, and human sustainable development [4]. The emergence of new AMR phenotypes and new multi-antimicrobial-resistant (MAR) bacteria has increased the complexity of this problem. Current projections indicate that AMR could lead to approximately 1·91 million annual deaths attributable to AMR globally by 2050, with an additional 8·22 million deaths annually associated with AMR [5].

The interactions of humans, animals, plants, and environmental systems are intricately connected to the dynamics of AMR. The emergence and dissemination of AMR are influenced by key factors, such as agriculture, food production, healthcare practices, environment, socioeconomic development, and geographical climate changes. These interconnected factors are closely intertwined with ecosystem development [6]. Conventional single-level and single-perspective approaches have proven inadequate for the effective prevention and control of AMR. To effectively address this issue, it is necessary to use multidisciplinary and multisectoral cooperation approaches that encompass human, animal, and environmental dimensions. The “One Health” approach offers a comprehensive and systematic framework for tackling AMR from a multidimensional and multifaceted perspective [7].

The World Health Organization (WHO), Food and Agriculture Organization of the United Nations, and Office International des Epizooties (International Organization for Animal Health), along with other international organizations, have highlighted the importance of adopting “One Health” to tackle AMR [8]. “One Health” emphasizes establishing a collaborative and multifaceted system to curb AMR. This system includes populations, animals, agriculture, the environment, and food to interrupt the emergence and rapid spread of bacterial AMR. This review discusses the mechanisms of AMR and the contributors to its generation and spread. Additionally, it also examines potential strategies for collaborative prevention and control of AMR across various institutions, disciplines, and regions.

## 2. Antimicrobial Consumption

The global rise in AMR is predominantly linked to selective pressures arising from improper use, overuse, or misuse of antimicrobials in humans and animals [9]. Consequently, antimicrobial consumption in human healthcare and veterinary sectors merits focused attention. Major multidrug-resistant bacteria from clinical and veterinary points of view are shown in Table 1. China has emerged as a top hot spot for antimicrobial use, maintaining the world’s highest consumption of both human and veterinary antimicrobials [10,11].

The intensity of antibiotic consumption in human medicine is typically quantified using defined daily doses per 1000 inhabitants per day (DID), a standardized metric for comparing antimicrobial utilization across populations and regions. In 2020, China’s antibiotic use measured 10.51 DID [12], lower than the European Union’s 16.4 DID [13]. The antibiotic use in the United States exceeds 20 DID [14]. While lower DID values typically indicate stringent antimicrobial stewardship policies and effective healthcare regulation, they may paradoxically reflect accessibility challenges in low-income regions.

The use of antimicrobials in animals has been practiced for several decades, primarily for disease prevention, growth promotion, and therapeutic purposes in livestock production. For the application of antibiotics in animal culturing, China used 84,240 tons for the total production of 87.76 million tons of meat products in 2013, while the United States used 14,600 tons for a total of 42.62 million tons of meat products in 2012 [15]. China’s animal antibiotic use intensity is 2.8 times as much as that of the United States [16]. In 2020, China’s antibiotic consumption in food-producing animals totaled 32,776.30 tons, corresponding to an intensity of 165 mg/kg [11]. In 2023, the veterinary pharmaceutical consumption in the European Union was recorded at 45.1 mg/kg. Notably, this figure encompasses antibacterials, antiprotozoals, antifungals, and anti-infectives reported by certain European nations [17]. However, it should be emphasized that since antibiotics constitute the predominant component of total antimicrobial consumption, this measurement discrepancy exerts a limited impact on the overall conclusions.

Globally, the consumption of antimicrobials in animals exceeds that in humans [18]. Approximately 50% of domestically produced antimicrobials are allocated to food animals. In the United States, over 80% of total antimicrobial consumption is directed toward food animals [9]. Unlike human medicine, where patients are treated individually with low doses of antimicrobials, animal producers must use mass medication since it is impractical to treat each animal in a group that consists of hundreds to tens of thousands [9]. Even if antimicrobial usage in animals and humans demonstrates comparable relative quantities per biomass unit, the substantially greater total animal biomass translates to higher probabilities of AMR mutation emergence in animal reservoirs [18]. This underscores the importance of adopting a One Health approach to understand how AMR impacts both animal and human ecosystems.

**Table 1:** Major Antimicrobial-Resistant Bacteria in Humans from Clinical and Veterinary Points of View.

Major Antimicrobial-Resistant Bacteria	Antimicrobial Resistance	Resistance Rates (%)		Resistance Genes	Reference(s)
		Human Clinical Points of View	Veterinary Points of View		
*Staphylococcus aureus*	Methicillin Resistance	29.6 (China); 15.8 (Europe); 38.1 (the United States)	7.6 (retail meats in the United States); 7.3 (raw meat in China)	*mecA*, *mecC*, *fem*	[19,20,21,22]
*Escherichia coli*	Extended-Spectrum β-Lactamases (ESBL)	13.3 (the United States); 53.5 (China); 18.5 (Europe)	0.9 (broilers in Europe); 69.3 (pigeons in China); 3.1 (companion animals in the United States)	*bla*_TEM_, *bla*_SHV_, *bla*_OXA_, *bla*_CTX-M_	[23,24,25,26]
	Carbapenem Resistance	0.3 (Europe); 1.0 (the United States); 2.1 (China)	4.9 (chickens in China); not detected (chickens in the United States)	*bla*_TEM_, *bla*_SHV_, *bla*_OXA_, *bla*_CTX-M_, *bla*_KPC_	[19,20,27,28,29,30]
	Colistin Resistance	1.3 (China); 0.3 (Europe); 0.3 (the United States)	17.0 (chickens in China); 0.1 (retail chickens in the United States)	*mcr*, *pmrAB*, *mgrB*, *phoPQ*	[19,20,27,28,29,31]
*Klebsiella pneumoniae*	ESBL	11.8 (the United States); 21.8 (China); 35.5 (Europe)	76 (mink in the United States); 44.5 (overall animal in Africa); 33.7 (overall animal in Asia)	*bla*_KPC_, *bla*_SHV_, *bla*_OXA_, *bla*_CTX-M_, *bla*_TEM_	[23,32,33,34,35,36]
	Carbapenem Resistance	13.3 (Europe); 10.8 (China); 24.6% (the United States)	3.8 (pets in China); not detected (poultry meat in Greece)	*bla*_OXA_, *bla*_KPC_, *bla*_VIM_, *bla*_IMP_, *bla*_NDM_	[19,20,37,38,39]
*Pseudomonas aeruginosa*	Carbapenem Resistance	18.6 (Europe); 14.2 (the United States); 18.2 (China)	13.5 (pets in China); 23.1 (shellfish in Croatia); not detected (horses, cows, and dogs in France)	*bla*_VIM_, *bla*_GES_, *bla*_NDM_, *bla*_KPC_	[19,20,27,37,40,41,42]
*Acinetobacter baumannii*	Carbapenem Resistance	69.5 (Europe); 71.5 (China); 45.7 (the United States)	not detected (bovine in Germany); 17.9 (raw meat in Iran)	*bla*_VIM_, *bla*_IMP_, *bla*_NDM_, *bla*_KPC_	[27,43,44,45,46]
*Enterococcus faecium*	Vancomycin Resistance	5.1 (China); 19.8 (Europe); 67.3 (the United States)	not detected (livestock in the United States); 0.4 (cats and dogs in China)	*VanA*, *VanB*, *VanC*, *VanD*, *VanE*, *VanM*	[19,20,47,48,49]
*S* *almonella*	Fluoroquinolone Resistance	21.8 (Europe); 0.6 (the United States); 26.6 (*Salmonella* Enteritidis in China); 19.7 (*Salmonella* Typhi and *Salmonella* Paratyphi in China)	74.0 (ducks and wild geese in China); 14.9 (pigs in the European Union); 4.1 (market swine in the United States)	*gyrA*, *gyrB*, *parC*, *parE*	[24,29,50,51]

* The term “not detected” indicates that no isolate exhibiting antimicrobial resistance was identified in this investigation.

## 3. Drivers of AMR

The essence of AMR emergence is the adaptive evolution of bacteria to external selection pressure [52], which is a relatively lengthy process for the evolution of bacterial AMR in nature [53]. However, for almost the past 100 years, overuse of antimicrobials has greatly accelerated this process, resulting in difficulty in effectively treating bacterial AMR globally. The emergence and spread of AMR are due to the interplay of “intrinsic determinants” and “extrinsic drivers”. Intrinsic determinants include antimicrobial resistance genes (ARGs) and mobile gene elements (MGEs) in the bacterial genome [54]. MGEs include plasmids, transposons, integrons, etc., which determine the potential ability of ARGs to spread. Extrinsic drivers include both direct and indirect factors. The direct factors are the selective pressure exerted by chemicals in the ecological niches, such as antimicrobials, heavy metals, disinfectants, etc. [55]. The indirect factors include social (e.g., national antimicrobial policy, environmental protection policy, etc.), economic (e.g., intensive farming density, the price of meat products, etc.), pharmaceutical (e.g., the price of antimicrobials, the mode of hospital-acquired infectious disease control, etc.), hygiene (e.g., food hygiene, environmental sanitation, etc.), and climate (e.g., temperature and humidity, environmental pollution, high winds, and heavy rains, etc.) contributors. Key Drivers of AMR are shown in Table 2.

### 3.1. The Intrinsic Determinants of AMR

**Figure 1:**
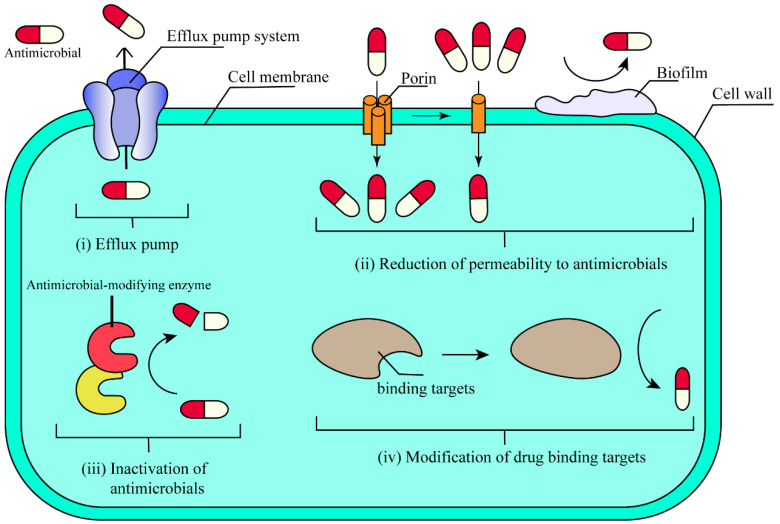
There are multiple mechanisms by which bacteria can develop resistance to antibiotics, including (**i**) efflux pump, (**ii**) Reduction in permeability to antimicrobials, (**iii**) inactivation of antimicrobials, and (**iv**) modification of drug binding targets.

ARGs enable the microorganism to exhibit AMR, leading to reduced efficacy of antimicrobial treatments [56]. Generally, the acquisition of AMR usually results from changes that directly or indirectly affect the drug–target interaction. There are four common mechanisms (Figure 1). (i) Efflux pump: Bacteria construct an efflux pump system using efflux, fusion, and outer membrane channel proteins. This efflux pump system excretes intracellular antimicrobials and reduces the concentration of intracellular antimicrobials to achieve AMR [57]. (ii) Reduction in permeability to antimicrobials [58]: Bacteria can regulate the concentration of intracellular antimicrobials through porin and biofilm, preventing the antimicrobials from binding to the target. Changes in the number of porins and their selectivity or function can limit the uptake of antimicrobials into the cell. Biofilm reduces the penetration of antimicrobials and the bacterial metabolism or growth rate to produce AMR [59]. (iii) Inactivation of antimicrobials [60]: Bacteria can produce modification enzymes and hydrolases that destroy antimicrobials or render them ineffective. Through this mechanism, antimicrobials can fail or be destroyed before they act on the target organism. (iv) Modification of drug binding targets: Usually, antimicrobials bind to their targets with extremely high specificity. Subtle changes in the binding target can seriously affect the binding of antimicrobials [61].

In bacteria, the distinctions between different bacterial species are not always clear-cut or strictly defined. Low-fidelity species boundaries drive widespread horizontal and vertical ARGs transfers, via either heterologous or homologous recombination [96]. Vertical gene transfer (VGT) is the transmission of ARGs from bacteria to their offspring by division and reproduction. During this process, there is a possibility of mutation in the genes, which could produce new ARGs. The mutation rate of genes is generally in the range of 10^−10^~10^−6^ mutations per nucleotide/year in the natural state. Hypermutators exist in bacteria, which can have mutation rates more than 1000 times higher than normal [97]. The formation of super mutants is mainly due to mutations in the methyl mismatch repair system. Horizontal gene transfer (HGT) is an important mechanism for bacteria to acquire exogenous ARGs from environmental bacteria. There are three main mechanisms of HGT [62]: (i) Conjugation, which involves establishing a protein channel between the donor bacteria and the recipient bacteria through a sex pilus. This channel offers a way to transfer ARGs. MGEs can serve as carriers of ARGs. Most ARGs are located on MGEs, with only a very small portion located on the genome. Therefore, MGEs are the major genetic basis for the transmission of ARGs across different bacterial species. (ii) Transformation, which is a mechanism by which a bacterium ingests and integrates foreign DNA fragments from the surrounding environment. After these fragments are integrated into the bacterial genome, the recipient bacterium can express certain AMR traits. (iii) Transduction, which is a process by which phages transfer genes between recipient and donor bacteria. Phages carry the genes of the host after they invade the host bacteria and then integrate the genes of the former host into the genome of the new host, leading to cross-species transmission of ARGs.

### 3.2. Extrinsic Drivers of AMR

#### 3.2.1. Environmental Stress Factors

Bacteria often adapt to harsh environments by altering gene expression or acquiring new ARGs [98]. Environmental stressors include antimicrobials, heavy metals, disinfectants, etc.

In clinical medicine, many treatments (e.g., joint replacement, cancer chemotherapy, and organ transplantation) use antimicrobials to inhibit infections. In animal husbandry, antimicrobials are used to prevent and control animal diseases and improve breeding efficiency and product quality [63]. However, antimicrobial misuse often results from over-reliance on antimicrobials or incorrect assessment of antimicrobial dosage [64]. Besides, an irrational combination of multiple antimicrobials reduces the antimicrobial effect, increases the side effects of antimicrobials, and enhances AMR. In addition, humans and animals can excrete antimicrobials and their metabolites into the environment [65]. These substances would accumulate in the environment and exert pressure on environmental bacteria. All these factors increase the likelihood of survival and reproduction of bacteria that develop or acquire ARGs through VGT or HGT. This, in turn, leads to the spread of ARGs among bacterial populations, leading to AMR and increasing the difficulty of the treatment of infections, which could have been otherwise controllable by antimicrobials, turning AMR into a major public health concern.

Heavy metals are an important source of environmental stress for bacteria. These substances are not easily biodegradable, and they persist and remain in the environment. Heavy metals can directly or indirectly have toxic effects on bacteria [66]. The direct toxic effects of heavy metal ions, such as mercury, silver, and arsenic salts, are mainly manifested through binding to the protein-SH group of bacterial enzymes, rendering them inactive [67]. Also, heavy metal ions carry a positive charge, and bacterial cell membranes carry a negative charge. Thus, heavy metal ions can adhere to the cell membrane through coulombic forces and lead to a reaction with the peptidoglycan in the cell wall, which can cause damage to the bacteria’s internal components or disrupt their production. As a result, the cell wall can be destroyed, the cytoplasm leaks out, ultimately resulting in bacterial death. The indirect toxic effects of heavy metals mainly include the following: (i) heavy metal ions can kill bacteria by activating the surrounding oxygen to produce reactive oxygen species and free radicals; (ii) heavy metals can bind antimicrobials to form complexes that enhance the activity of antimicrobials, which can stimulate bacteria to develop co-resistance mechanisms to antimicrobials, including synergistic resistance, cross-resistance, and co-regulation. Co-resistance indicates the presence of the same genetic element carrying different ARGs. Cross-resistance is the mechanism by which bacteria can resist the toxic effects of both heavy metals and antimicrobials. The bacterial efflux pump system is a typical cross-resistance mechanism that can regulate the concentration of heavy metals and antimicrobials in the bacterial cell. Co-regulation is the synergistic response of bacteria in response to antimicrobial and heavy metal-induced co-stress. Bacteria can increase the transcriptional–translational activity of certain ARGs and enhance AMR under heavy metal-induced stress [68]. It has been reported that heavy metal-induced stress has a significant effect on both the presence of ARGs and HGT events [69].

Disinfectants can prevent and control the occurrence of bacterial-related diseases by inhibiting or killing bacteria. Thus, they are widely used in the environmental disinfection of public places and medical institutions. However, irrational use of disinfectants can lead to bacterial resistance to disinfectants and antimicrobials. Bacteria exposed to sub-inhibitory or sub-lethal concentrations of disinfectants show increased tolerance to disinfectants and antimicrobials [70]. Bacteria can become resistant to disinfectants in multiple ways, including by altering their targets, producing specific enzymes, reducing membrane permeability, expressing efflux pumps, and forming biofilms. The mechanisms of cross-resistance and co-resistance of bacteria to disinfectants are similar to those of heavy metals. The misuse of disinfectants further worsens the state of AMR and causes greater harm than bacterial AMR itself. Co-resistance to disinfectants and antimicrobials poses greater challenges and difficulties in the treatment of some bacterial diseases. For example, during the COVID-19 pandemic, large quantities of disinfectants were used for prophylactic disinfection of surfaces and items to prevent the spread of the SARS-CoV-2 virus. The sustained use of disinfectants on a large scale may accelerate the production and spread of disinfectant-resistant and antimicrobial-resistant bacteria [71].

#### 3.2.2. Social Factors

From the government’s perspective, it is crucial to develop policies related to the rational use of antimicrobial medications and environmental protection to control bacterial AMR. Epidemiological studies have found that the optimization of antimicrobial use policies and antimicrobial-specific environmental protection policies is crucial for controlling the occurrence and spread of bacterial AMR [72]. A study showed that antimicrobial misuse was worse in those countries without a scientific antimicrobial use policy. The effective implementation of antimicrobial use policies can significantly reduce bacterial AMR [73]. In addition, the entry of a large amount of antimicrobials and their metabolites into the environment worsens the AMR problem. Annually, China discharges over 50,000 tons of antimicrobials into both aquatic and terrestrial ecosystems, thereby engendering an antimicrobial selection pressure within these environments that markedly surpasses that observed in other nations [74]. For instance, when considering the overall concentration of antimicrobials present in riverine systems, an examination of 58 river basins within China has disclosed the utmost concentration of diverse antimicrobials to escalate to as much as 7560 ng/L, with the mean concentration standing at 303 ng/L. By comparison, such concentrations are apparently lower in other countries, evidenced by measurements of 9 ng/L in Italy, 20 ng/L in Germany, and 120 ng/L in the United States. This is related to the lack of policy on antimicrobial medication discharge and the extensive use of antimicrobials in agriculture. Strict discharge regulations are necessary to reduce antimicrobial pollution in the environment [75]. If there is a lack of strict regulation of the main sources of environmental antimicrobial emissions, such as pharmaceutical factories, medical institutions, and farms, it will be extremely difficult to maintain the level of antimicrobials in the environment below acceptable levels. It has been confirmed that unregulated wastewater discharge is one of the main reasons for the significant increase in antimicrobial concentrations in rivers and groundwater [76]. These pollutants may eventually lead to increased resistance of bacterial microbiota to antimicrobials. Therefore, in overviewing the global situation, it is particularly important to promote and implement rational antimicrobial use policies and environmental protection policies to effectively prevent and control bacterial AMR.

#### 3.2.3. Economic Factor

Economic factors such as the price of meat and agricultural production mode play a significant role in stimulating AMR. There has been a debate that the decreasing prices of meat products have led to the rise in intensive farming. When meat prices drop, farmers tend to cut costs, pushing them towards intensive farming. The characteristics of intensive farming include the use of large amounts of antimicrobials to optimize production efficiency, such as to promote animal growth and prevent disease [77]. Scholars from various countries have raised concerns regarding the use of antimicrobials in intensive farming. For example, a study showed that the popularity of intensive farming has led to a significantly increased use of antimicrobials [78]. The large-scale use of antimicrobials not only increases the potential presence of their residues in food and the environment but also leads to a greater probability of AMR generation. In recent years, scientific interest in antimicrobial pollutants released from farms and the potential for these pollutants to trigger AMR has increased. Antimicrobial residues put pressure on bacterial microbiota in wastewater treatment facilities [79] or the natural environment and then induce the emergence of antimicrobial-resistant bacterial populations [80]. Therefore, from an economic perspective, the problem of bacterial AMR cannot be ignored amidst decreasing meat prices and intensive farming practices.

#### 3.2.4. Medicinal Factors

Hospitals are the epicenter for the development of AMR [55]. Despite clinicians’ specialized training in antimicrobial stewardship, inappropriate prescribing persists through both non-indicated usage and suboptimal therapeutic application. UK surveillance data indicate approximately 50% of antimicrobial prescriptions demonstrate suboptimal clinical appropriateness [81]. The critical role of Intensive Care Units (ICUs) in accelerating AMR development warrants particular attention. ICU patients typically present with multiple comorbidities, frequent hospitalization histories, prolonged ICU stays, multiple prior antibiotic courses, and complex pathophysiology. These are factors that may lead to suboptimal dosing (both under- and over-administration) and create ideal conditions for AMR emergence [82]. These risks are compounded by some key ICU-specific factors: the routine use of invasive medical devices, heightened exposure to broad-spectrum antibiotics, and the intensive nature of therapeutic interventions. The convergence of microbial colonization risks from invasive procedures, selective pressure from extensive antibiotic use, and compromised host defenses establishes an ecological niche particularly conducive to ARGs propagation and pathogen evolution [83]. Cross-contamination represents a critical pathway for AMR development in hospital settings, where antimicrobial-resistant bacteria transfer between patients, medical equipment, and healthcare personnel. Inpatient populations, particularly immunocompromised patients, are frequently susceptible to antimicrobial-resistant bacterial infections. Both improperly isolated patients with antimicrobial-resistant bacterial infections and asymptomatic carriers serve as transmission reservoirs, while healthcare workers may themselves become colonization hosts and novel transmission sources through inadequate contact precautions and disinfection protocols.

At the community level, the development of AMR is driven by synergistic interactions among multiple factors [84]. Firstly, antibiotic misuse and non-prescription access constitute a core issue: patients frequently self-prescribe antibiotics for non-bacterial infections or prematurely discontinue treatment courses, creating selective pressure that favors the emergence of resistant mutants under suboptimal antibiotic concentrations. Secondly, inadequate healthcare infrastructure exacerbates this trend. Primary care facilities, lacking rapid pathogen diagnostics and real-time antimicrobial resistance surveillance systems, predominantly rely on empirical broad-spectrum antibiotic therapy, amplifying resistance risks. Moreover, complex human–animal–environment interactions within communities significantly facilitate the dissemination of ARGs. Firstly, antibiotic misuse and non-prescription access constitute a core issue: patients frequently self-prescribe antibiotics for non-bacterial infections or prematurely discontinue treatment courses [99], creating selective pressure that favors the emergence of resistant mutants under suboptimal antibiotic concentrations. Secondly, inadequate healthcare infrastructure exacerbates this trend. Primary care facilities, lacking rapid pathogen diagnostics and real-time antimicrobial resistance surveillance systems, predominantly rely on empirical broad-spectrum antibiotic therapy, amplifying resistance risks [100]. Moreover, complex human–animal–environment interactions within communities significantly facilitate the dissemination of ARGs [101].

Undesirable pharmaceutical practices are a major factor in increasing AMR [85]. Some pharmaceutical companies manufacture low-quality antimicrobials to increase profits. These substandard drugs may have insufficient dosage or unstable active ingredients. Infected bacteria would undergo mutational evolution due to the selective pressure produced by the subtherapeutic amounts of antimicrobials. Such amounts cannot kill all bacteria, but can result in the development of AMR and epibiotic bacterial transmission. Studies have also found that prolonged overuse of antimicrobials induces bacteria to develop AMR, resulting in challenges for patients and the healthcare system.

#### 3.2.5. Health Factors

Food hygiene and environmental sanitation are also important factors in the development of AMR. Antimicrobial residues in foods greatly increase the potential development of AMR [86]. Foods of animal origin have antimicrobial and/or their metabolite residues due to the misuse of antimicrobials at the farm level [87]. This leads to foodborne exposure to antimicrobials and increased antimicrobial-resistant bacteria in human intestinal microbiota. Due to the long-term use of veterinary antimicrobials, MAR bacteria emerge in the intestinal tract of animals. These MAR bacteria are excreted in feces and enter the farmland with organic manure application, leading to the emergence and spread of bacterial AMR in the food supply chain. Currently, farm animal organic manure is often applied as a base fertilizer in green vegetable cultivation. Moreover, antimicrobial-resistant bacteria and ARGs can enter plants through polluting soil, which can further enhance the AMR of endophytic plant bacteria [88], thereby increasing the risk of bacterial AMR exposure in humans from food intake.

#### 3.2.6. Climatic Factors

Temperature affects bacterial growth and alters the transfer of genes, including ARGs. The spread of AMR can be accelerated by temperature and climate change [89]. Elevated temperatures can lead to increased AMR to certain antimicrobials [90]. In addition, temperature affects the physiological response of organisms. In recent years, the rise in global temperature has caused changes in the living environment. These changes have facilitated the spread of pathogens, thus increasing the incidence of infectious diseases. As a result, more antimicrobials will be required to control infectious diseases, which will ultimately worsen AMR.

Atmospheric particulate matter carries a large number of ARGs [91]. Air contaminated with atmospheric particulate matter is a vector for AMR spreading. The spread of antimicrobials and antimicrobial-resistant bacteria may also be enhanced by high winds, which facilitate the spreading of bacteria, ARGs, and antimicrobials in the environment and among populations. In livestock farming, manure handling, sewage treatment, and sites teeming with bacteria, high winds, and rainfall can rapidly spread and dramatically enlarge antimicrobial-resistant bacterial contamination.

#### 3.2.7. Ageing Factors

Global demographic trajectories demonstrate a marked escalation in geriatric population metrics across both absolute and proportional dimensions. Empirical data from the United Nations Population Division revealed that the cohort aged ≥65 years reached 770 million in 2022, with projections indicating this figure will surpass 1.5 billion by mid-century (2050) [102]. This demographic shift is particularly pronounced in high-income nations, where longitudinal survival analyses indicate the probability of neonates surviving to age 90 has escalated from 4.8% in 1950 to 26.7% in the contemporary era [92]. The geriatric population exhibits distinct pathophysiological characteristics that synergistically potentiate AMR development through multiple mechanisms [93]. Age-related physiological decline—including diminished cellular regenerative capacity, impaired organ function, and decelerated metabolic processes—induces systemic immunosenescence. This biological vulnerability predisposes older adults to heightened susceptibility to infections, necessitating heightened antimicrobial utilization for both therapeutic and prophylactic purposes. Such frequent antibiotic exposure amplifies selective pressure for resistant pathogen strains, thereby accelerating AMR propagation. Concurrently, the high prevalence of chronic comorbidities in aging populations introduces iatrogenic AMR risks. A study found that five non-antibiotic drugs, including anti-inflammatory drugs (ibuprofen, naproxen, and diclofenac), lipid-lowering drugs (gemfibrozil), and β-blockers (propranolol), promoted transmission of ARGs through bacterial transformation [94]. In addition, some chronically ill patients who are susceptible to infection by pathogens are thought to be reservoirs of antimicrobial-resistant pathogens [95]. Infectious diseases, chronic illnesses, and other medical needs drive older adults to make frequent trips to places at high risk for the spread of antimicrobial-resistant pathogens, such as health care facilities or long-term care facilities.

## 4. Transmission and Evolution of Antimicrobial-Resistant Bacteria

### 4.1. Transmission of Antimicrobial-Resistant Bacteria

Several studies have identified possible mechanisms for the generation and spread of antibacterial-resistant bacteria and ARGs, including microbiota changes [103], acquisition of ARGs [104], and large-scale dispersal [105]. Dominant microbiota changes may lead to the spread of antimicrobial-resistant bacteria. Also, due to selective pressure from the external environment, such as antimicrobials, heavy metals, and disinfectants, antimicrobial-resistant bacteria may have a greater survival advantage than sensitive bacteria, after replacing sensitive bacteria as the dominant microbiota. Animals also play an important role in the spread of antimicrobial-resistant bacteria [106]. In livestock farming, the use of antimicrobials may lead to an increase in antimicrobial-resistant bacteria in the microbiota. The environments fostered within the confines of the livestock industry, where animals are densely housed, constitute optimal breeding grounds for the proliferation of antimicrobial-resistant bacteria. Within these settings, animals are not only exposed to such bacteria but also serve as conduits for the dissemination of these strains through various vectors, including excreta, water, and feed. The intimacy of contact between humans and livestock markedly elevates the probability of antimicrobial-resistant bacterial transmission [107]. Individuals in close association with livestock, such as farm workers, veterinarians, and farmers, are particularly vulnerable to infections by these resistant strains, primarily through direct contact [108]. Furthermore, the transmission of antimicrobial-resistant bacteria to humans via the food chain represents a significant risk. The consumption of contaminated animal products, encompassing meat and dairy items, facilitates humans’ ingestion of antimicrobial-resistant bacteria, potentially leading to infection. The role of pet ownership in amplifying the spread of antimicrobial-resistant bacteria is also noteworthy [109]. Pets may consume antimicrobial-resistant bacteria through contaminated pet food [110], and the misuse of antimicrobial medications or exposure to contaminated environments in medical facilities may result in pets being infected with antimicrobial-resistant organisms. The close interactions between pets and their hosts at home, such as licking skin and sharing sleeping spaces, increase the likelihood of transmitting antimicrobial-resistant bacteria from pets to their human companions [111]. Transmission of ARGs is another pathway to generate AMR. The microbiota composition may remain stable, but sensitive bacteria may become antimicrobial resistant by acquiring ARGs through HGT under external survival pressures. This dramatic gene transfer pathway may have accelerated the spread of AMR.

**Figure 2:**
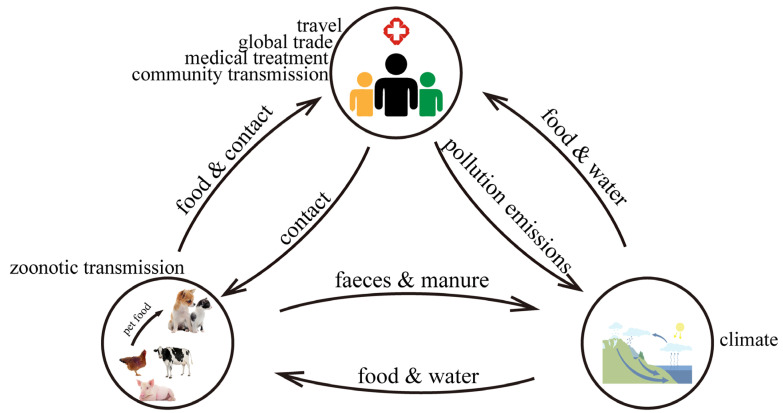
Schematic of potential transmission pathways of antimicrobial resistance genes between human, environmental, and animal reservoirs.

Man-made large-scale diffusion is also an important pathway for the spread of antibacterial-resistant bacteria and ARGs, such as animal/animal food transportation, population movement [112], etc. Such spreading may expand the geographic distribution of antimicrobial-resistant bacteria and ARGs. In this pathway, potential “object-to-person” transmission (Figure 2) may lead to the spread of antimicrobial-resistant bacteria and ARGs in the population, which may make the prevention of infectious diseases more difficult. All the above transmission types may affect microbial community stability and ecosystem balance in certain regions [113]. Although some research progress has revealed the contributors to the emergence, spread, and evolution of antimicrobial-resistant bacteria and ARGs, some underlying mechanisms and their influence remain unclear [114]. A better understanding of these mechanisms could improve public health strategies to prevent AMR.

### 4.2. Evolution of Antimicrobial-Resistant Bacteria

Human activities and the development of medicine and health behaviors have profoundly altered the bacterial survival environment throughout history, which in turn affects the microbiota and changes AMR [115]. Studies have indicated that *Enterococcus faecium* has undergone two major population differentiation events during its evolution, both of which are closely related to the changes in human activities. Bayesian evolutionary analysis of *E. faecium* genomes showed two major branches, namely the human-derived and animal-derived ones, which split 2776 ± 818 years ago. This split almost coincided with the rise in human domestication of wild animals and urban settlement, suggesting that human activities may have caused the divergence [116]. About 74 ± 30 years ago, *E. faecium* of human origin diverged a second time into a hospital-endemic AMR evolutionary branch and a community-endemic non-AMR evolutionary branch. The period of occurrence of this divergence coincided with the widespread use of antimicrobials for anti-infective treatment in humans and animals. These findings emphasize that human activities, medicines, and health behaviors apparently drive the evolution and divergence of the microbiota and cause the emergence of AMR.

## 5. Impact of AMR on Humans

### 5.1. Human Health

AMR is a threat to human health that could hamper the control of many infectious diseases and dramatically set back modern medicine. The massive and irregular use of antimicrobials has accelerated the arrival of the “post-antimicrobials era”, meaning that the sensitivity of pathogenic bacteria to antimicrobials has been decreasing, resulting in a significant reduction in the effectiveness of clinical infection treatment. Things as common as strep throat or a child’s scratched knee could once again kill [9]. AMR makes infections harder to treat, leading to increased disease severity, prolonged illness, and higher mortality rates. It makes medical procedures and treatments, such as surgery, caesarean sections, cancer chemotherapy, and organ transplantation, much riskier. Patients undergoing these procedures may face a higher risk of postoperative infections due to the presence of antimicrobial-resistant pathogens, which can complicate their recovery and increase the likelihood of complications. In addition to the direct impact on individual health, AMR also has significant indirect consequences for public health and healthcare systems. The spread of AMR can lead to increased healthcare costs as more expensive and intensive treatments are required to manage AMR infections [117]. This places a greater burden on healthcare systems and can limit access to effective medical care for patients. World Bank projections indicate that maintaining current trajectories could result in AMR-related healthcare expenditures of up to 1 trillion USD by 2050.

### 5.2. Food Security and Safety

As a result of the increasing human demand for animal protein, intensive farming practices have led to the widespread use of antimicrobials as veterinary drugs and growth promoters since the 1950s. Although this trend has had a positive impact on the elevated supply of animal protein, it has also led to the widespread spread of antimicrobial-resistant bacteria and ARGs in farming systems. Farmed animals and their products (meat, eggs, and milk) as well as production sites (such as farms and slaughterhouses) have become reservoirs and dispersers of antimicrobial-resistant bacteria/ARGs [107]. Increased AMR may lead to more frequent illnesses in farm animals and reduce the effectiveness of conventional antimicrobial treatment. The death or slowing of the growth of sick animals in the livestock sector may reduce productivity and profitability, thus impacting food security and prices. Therefore, a large investment by official organizations in studying the impact of AMR on food prices and supply is necessary.

In recent years, the China National Center for Food Safety Risk Assessment (CFSA) has engaged in an extensive series of investigations focusing on the emergence and dissemination of antimicrobial-resistant bacteria across animals, food products, environmental contexts, and human populations, grounded in the “One Health” paradigm [118]. Collaboratively, CFSA and the Beijing Center for Disease Control and Prevention undertook a comprehensive cross-sectional analysis concerning AMR associated with significant foodborne pathogenic bacteria found in fresh livestock, poultry, and aquatic commodities marketed within Beijing. This investigation elucidated that the AMR prevalence for *Escherichia coli* against polymyxin B and colistin stood at 8% and 10%, respectively. Corresponding figures for *Salmonella* were 5.5% and 6%, for *Klebsiella pneumoniae*, 2% and 4%, and for *Pseudomonas aeruginosa*, 1% and 3%, respectively. Through the lens of functional genomics, analysis revealed the presence of the polymyxin resistance gene *mcr-3* in 12 isolated strains, representing a detection frequency of 7.10%. Predominantly, E. coli strains derived from chicken sources were found to carry the high-level polymyxin resistance gene *mcr-1*. Furthermore, plasmid typing discerned that the *mcr-3* gene predominantly resided on plasmids (eight out of twelve instances, accounting for 66.7%), with IncI2 (six out of eight, or 75.0%) and IncY (two out of eight, or 25.0%) identified as the primary plasmid categories.

Utilizing advanced methodologies in machine learning and genomics, researchers have elucidated a sophisticated network of interactions encompassing environmental factors, microbial communities, and AMR within several broiler farms and slaughterhouses. An aggregate of 145 prevalent mobile ARGs was identified during the breeding and slaughtering phases. Furthermore, comprehensive analyses revealed the presence of 233 ARGs in conjunction with 186 microbial species within the intestines of broilers. These ARGs exhibited correlations with AMR levels in bacteria such as *E. coli*, which were isolated from broiler intestines. Additionally, 38 ARGs associated with resistance mechanisms in bacteria pathogenic to humans were detected. Employing machine learning techniques facilitated the identification of ARGs and their mutations linked to *E. coli* and *Salmonella*. The research also indicated a significant correlation between environmental parameters, such as temperature and humidity, and the distribution of ARGs. This integrated approach underscores the complex interplay between environmental conditions, microbial ecology, and the propagation of AMR, highlighting the critical need for comprehensive strategies in addressing AMR challenges [119].

In China, chickens have been found to have high levels of contamination with MAR bacteria, such as *Salmonella*, fluoroquinolone- and macrolide-resistant *Campylobacter*, and *E. coli* carrying ESBL [120]. Utilizing *Salmonella*, the predominant foodborne pathogen, as a case study, it becomes evident that the rates of AMR and MAR to fourteen common antimicrobial agents are alarmingly divergent across regions. In the European Union, these rates are reported at 29.3% and 17.7%, respectively. Contrastingly, in China, the figures soar to 82.5% and 63.8%. A particularly acute concern arises from the AMR profiles of *Salmonella* strains in China against frontline antimicrobials, such as third and fourth-generation cephalosporins and fluoroquinolones, which are critical for treating clinical infections [121]. This resistance trend portends a significant disease burden and underscores the looming threat of a post-antimicrobial era devoid of effective treatment options.

In a Chinese nationwide survey, antimicrobial susceptibility testing was conducted on 36,822 strains of pathogens responsible for foodborne diseases. A staggering 87.2% of these strains exhibited AMR to at least one antimicrobial, with a MAR rate of 66.5%. The highest resistance was noted for ampicillin, whereas imipenem maintained the lowest resistance rate. Variability in AMR rates across different serotypes was observed, with trend analysis revealing a decrease in resistance rates for gentamicin, nalidixic acid, ciprofloxacin, and polymyxin. Monitoring AMR patterns in clinically isolated foodborne pathogens is pivotal for deciphering trends in AMR, assessing the impact of AMR control strategies, and informing the clinical management of infections and precision in pharmacotherapy [122].

These antimicrobial-resistant bacteria cross-spread multi-directionally in farming, the environment, food, and people. The spreading leads to population exposure to the antimicrobial-resistant bacterial infection through a variety of ways. In contrast to the achievement assessment in the control of clinical antimicrobial use in China, there is still a lack of systematic assessment of the social risk and disease burden associated with exposure to antimicrobial-resistant bacteria/ARGs from farming. Therefore, the food safety field needs to make similar efforts to those made in the clinical medical field to give a more comprehensive and systematic assessment.

### 5.3. Healthy Carriers

Within the ambit of the One Health paradigm, the surveillance concerning the transmission of antimicrobial-resistant bacteria among asymptomatic individuals has been markedly overlooked [123]. Historically, in the annals of human infectious diseases, there exists a notable instance of an asymptomatic carrier facilitating the dissemination of a disease, epitomized by the case of “Typhoid Mary”. Asymptomatic carriers are capable of transmitting pathogenic bacteria and their associated AMR to vulnerable individuals, culminating in illness [124]. The Chinese Center for Disease Control and Prevention has undertaken the surveillance of healthy workers, adhering to the stipulations of the Food Safety Law of the People’s Republic of China and the Measures for the Administration of Preventive Health Examinations. Over a decade of continuous and methodical surveillance, researchers amassed and scrutinized 370,000 samples from asymptomatic workers to gauge the prevalence of *Salmonella* in their gastrointestinal tract, alongside conducting AMR and genomic epidemiological analyses. The findings disclosed a significant incidence of AMR among *Salmonella* strains harbored by individuals involved in food handling, with over 30% of these strains exhibiting MAR. An upward trajectory in AMR and MAR was observed in specific serotypes, including strains impervious to all primary antimicrobials conventionally deployed for the treatment of diarrheal diseases [125] (quinolones, third-generation cephalosporins, carbapenems, and polymyxins). Genomic sequencing and analyses pinpointed the predominance of these ARGs on plasmids, thus amplifying the risk of their transmission. Furthermore, the identification of the *mcr-1* gene variant in *Salmonella* strains isolated from asymptomatic individuals has been documented by both domestic and international media outlets, underscoring the public health peril engendered by the transmission of hyper-resistant genes by asymptomatic carriers [126]. Surveillance has registered a precipitous decline in the prevalence of the *mcr* gene among *Salmonella* strains carried by food handlers since 2018, coinciding with the implementation of China’s comprehensive ban in April 2017 on the utilization of polymyxins as growth promoters in agriculture. This development is indicative of China’s proactive and efficacious enforcement of the “Veterinary Drug Administration Regulations” promulgated by the Ministry of Agriculture.

### 5.4. Social Economy

AMR has a significant socioeconomic impact. The annual loss of productivity due to AMR in the United States has been estimated at $35 billion. Some international organizations have warned that AMR may worsen global poverty. In a 2017 World Bank report, it was projected that by 2050, AMR could push more than 28 million people into extreme poverty, especially in low- and middle-income countries. There are significant differences in pathogen AMR and antimicrobial consumption in countries with different income levels [127]. Low- and middle-income countries (LMICs) have relatively higher AMR rates of pathogenic bacteria and lower rates of antimicrobial consumption compared to high-income countries. Thus, in LMICs, the priority is to develop antimicrobial stewardship and infection control strategies to curb the emergence and spread of AMR pathogens. However, some LMICs lack adequate regulations and awareness about rational drug use. As a result, diseases caused by antimicrobial-resistant pathogens cannot be effectively treated, leading to a high disease burden. This problem can also further affect socioeconomic development. Moreover, agriculture and industry in these countries are highly dependent on intensive labor, but the lack of workers due to sickness can have a significant impact on the economy. AMR can also have a serious impact on animal health, affecting not only the farming industry but also the meat price. This would result in a lack of animal protein intake and lead to malnutrition. As predicted by the World Bank, AMR could have a significant impact on economic performance [128], especially in developing countries that are largely dependent on agriculture.

China has completely banned the use of antimicrobials in animal feed since 2020. This policy has brought a dramatic change in the method of livestock and animal food production. Far-reaching effects on social, economic, medical, and health aspects will also be brought by this policy in the future [129]. Therefore, based on the “One Health” concept, it is important to comprehensively evaluate the impact of this policy in China. Also, to improve healthcare, it is important to ban the use of antimicrobials in animal feed. As it can help reduce the risk of AMR exposure from the food chain and minimize the chances of humans consuming antimicrobials through food. In addition, banning the addition of antimicrobials to animal feed also means reducing the spread and accumulation of antimicrobial-resistant bacteria/ARGs in the environment, thus having a good effect on environmental health. Nonetheless, with respect to their efficacy, the majority of antimicrobials did not exhibit a significant decline in resistance trends. A select number of antimicrobials, those contingent upon specific ARGs such as polymyxin, displayed a reduction in resistance following their prohibition. The primary causation for this phenomenon resides in the cumulative selective pressure exerted by antimicrobials within the environmental milieu, engendering a formidable “environmental gene pool” replete with AMR. The extant antimicrobial stewardship strategies, in their current scope and implementation, are ineffectual against the pervasive and multifaceted ARGs characterized by broad-spectrum resistance and generalist tendencies. For instance, whilst China has proscribed the use of chloramphenicol in animal husbandry, it concurrently sanctions the use of florfenicol, a derivative of chloramphenicol. The overlapping resistance mechanisms between these two antibiotics render the prohibition of the former inconsequential. Consequently, the paramount and most efficacious strategy currently available in China involves the diminution of antimicrobial selective pressure across the entire environmental spectrum.

From a social perspective, this initiative aims to raise public awareness about healthy food, which will increase demand for safe and nutritious food. This, in turn, may lead to a change in production patterns within the food-related industry, thus encouraging more producers to prioritize the quality of their products. At the educational level, this will also strengthen public awareness of antimicrobials, reinforce public health consciousness, and reduce the blind use of antimicrobials.

## 6. Limitations and Prospects

Optimizing the use of antimicrobials is a major goal of WHO’s global action plan to combat AMR. The WHO introduced the Access, Watch, Reserve (AWaRe) classification framework for antibiotics in 2017, categorizing them into three tiers—Access, Watch, and Reserve—with the primary objective of optimizing antimicrobial stewardship and mitigating the emergence and spread of AMR [130]. Antimicrobials are categorized under the AWaRe classification to prioritize their use and combat AMR. Common agents include β-lactams (e.g., penicillins, cephalosporins) and sulfonamides (Access group); aminoglycosides, macrolides, fluoroquinolones, and lincomycins (Watch group); and peptides (e.g., polymyxins) and select reserve antimicrobials (Reserve group). Alarmingly, resistance is now emerging even in the Reserve group’s molecules, such as colistin [131] and meropenem [132], threatening the final line of defense against multidrug-resistant pathogens.

The emergence of AMR involves a vast number of bacterial populations and interactions between hosts and their environments. The evolutionary dynamics of AMR occur in various ecological niches. Although some studies have identified important ARGs and their transmission among bacteria through HGT and VGT mechanisms, the multifaceted knowledge of ARG sources, their diffusion pathways, speed of spread, and scope of influence remains unclear. Furthermore, although previous studies have revealed the AMR drivers, how to conduct effective interventions and management to further stop or slow down the emergence and spread of AMR remains an important challenge. In addition, many studies lack attention to the direct link between AMR and human health effects. This not only limits the applicability of research results but may also misestimate the role of research dimensions [133]. Therefore, there is a need to promote not only in-depth research on AMR in China but also interdisciplinary, cross-regional, and cross-border collaborations to overcome the shortcomings of focusing on only one perspective to deal with this public health problem. “One Health” takes a holistic systemic view from the perspectives of the sciences [134], humanities, and society. Integrating the relationships among human, animal, and environmental health prevents, predicts, monitors, and resolves the spread of antimicrobial-resistant bacteria, continuously balancing and optimizing the relationship between the three to promote multi-system health. Although the use of the precautionary principle is advocated to assess AMR risk, it is not feasible or sensible to eliminate all AMR reservoirs. Therefore, identifying causal factors in the transmission of AMR to humans is essential for the development of prioritized prevention and control strategies. Combining research strengths in ecology, microbiology, genetics, and bioinformatics [135], and using cutting-edge research tools to deeply explore the mechanisms of emergence and spreading of AMR and the influencing factors, provides the scientific basis and technical means for the development of effective strategies to curb AMR.

Current global AMR surveillance systems exhibit significant geographic disparities in data coverage and variable data quality. In many LMICs, AMR monitoring remains largely absent in primary care and community healthcare settings, resulting in incomplete and non-representative datasets [136]. A critical limitation lies in the sparse distribution of hospitals equipped with AMR diagnostic and reporting capabilities. These facilities predominantly serve critically ill patients who often receive prior antibiotic treatments, inadvertently skewing reported resistance rates toward more severe cases. Consequently, surveillance data from such settings may overestimate resistance levels for certain pathogen–drug combinations compared to broader populations [137]. Furthermore, insufficient diagnostic coverage and inadequate laboratory infrastructure hinder the accurate interpretation of AMR incidence variations [138]. Methodological inconsistencies—including divergent definitions of resistance, non-standardized sampling protocols, and variability in laboratory techniques—contribute to pronounced data heterogeneity [139]. This lack of harmonization undermines cross-regional comparability and reliability, complicating efforts to discern true resistance trends or evaluate intervention efficacy. Additional challenges arise during data integration and standardization. Aggregating AMR data from disparate sources (e.g., community-acquired vs. hospital-acquired infections) risks misclassification biases and methodological discordance [139]. For instance, merging datasets without adjusting for contextual differences in sampling frameworks or diagnostic criteria may introduce artifacts that distort epidemiological conclusions. Such limitations underscore the urgent need for unified surveillance protocols, capacity building in under-resourced regions, and interoperable data systems to enhance global AMR tracking accuracy and utility. The current health risk assessment system for AMR/ARGs is based solely on the pathogenicity of bacteria, the type of ARG, and whether it is transmitted genetically through mobile genetic elements to assign a risk level. There are few studies that assess the impact of actual transmission scenarios on the disease burden, population health, society, and economic development. Therefore, it is necessary to establish a multidisciplinary, multi-system, and cross-sectoral data-sharing platform that enables the exchange of multivariate data on the emergence, transmission, evolution, and distribution of AMR/ARGs in animals, environments, and populations. Multi-omics analysis, big data computing, machine learning [140], and data simulation are important tools to better understand and predict the intrinsic determinants and extrinsic drivers of AMR. A predictive and early warning system should be developed based on the assessment of the potential risks of antimicrobial-resistant bacteria/ARGs on population health and socioeconomics. In addition, it is necessary to build a risk prevention and control policy system for antimicrobial-resistant bacteria/ARGs based on the “One Health” concept, to maintain the balance between socioeconomic development and human health.

## Figures and Tables

**Table 2:** Key Drivers of Antimicrobial Resistance.

Driving Factor		Current Status	Causes	Reference(s)
Intrinsic determinants	Antimicrobial Resistance Genes	Antimicrobial resistance genes are widely distributed in bacterial populations, enabling resistance through enzymatic or structural adaptations.	Mutations and vertical gene transfer during bacterial replication.	[56,57,58,59,60,61]
	Mobile Gene Elements	Mobile gene elements act as carriers for antimicrobial resistance genes, facilitating rapid horizontal spread.	Conjugation, transformation, and transduction mechanisms.	[62]
Extrinsic drivers	Antimicrobial Misuse	Accumulation of antibiotic residues in the environment enhances bacterial resistance.	Overuse in healthcare and livestock, incomplete metabolism, and environmental discharge of antimicrobials.	[63,64,65]
	Heavy Metal Effects	Synergistic effects between heavy metals and antimicrobials promote co-resistance.	Industrial/agricultural pollution; bacterial efflux pumps resisting both heavy metals and antimicrobials.	[66,67,68,69]
	Disinfectant Misuse	Increased cross-resistance to disinfectants and antimicrobials.	Excessive disinfectant use during pandemics; bacterial adaptation via target modification or efflux pumps.	[70,71]
	Social Factors	Widespread antimicrobial misuse and weak regulatory policies.	Inadequate national antimicrobial policies; poor enforcement of environmental regulations (e.g., wastewater discharge).	[72,73,74,75,76]
	Economic Factors	Intensive farming practices amplify antimicrobial residues and resistant bacteria.	Declining meat prices drive antimicrobial-dependent farming; surging antimicrobial use in livestock.	[77,78,79,80]
	Medicinal factors	(1) Hospitals and communities promote the development of AMR. (2) Substandard antimicrobials create subtherapeutic selection pressure.	Inappropriate prescribing and cross-infection in hospitals drive AMR transmission, while ICU-specific interventions exacerbate AMR risks. Community antimicrobial misuse drives AMR, compounded by weak healthcare systems and human–animal–environment interactions. Production of low-quality antimicrobials; irrational prescribing practices (e.g., underdosing).	[81,82,83,84,85]
	Health Factors	Contaminated food/environment increases human exposure to resistant bacteria.	Antimicrobial residues in animal-derived products; manure fertilization spreading AMR to soil and crops.	[86,87,88]
	Climate Factors	Climate change accelerates AMR spread via airborne particles and extreme weather.	Rising temperatures enhance bacterial growth/gene transfer; storms disperse pollutants and ARGs.	[89,90,91]
	Aging Factors	The aging population has accelerated the development of AMR.	Older adults’ susceptibility to infections and high prevalence of chronic diseases lead to increased medical visits in AMR-prone settings and antimicrobial usage.	[92,93,94,95]
